# Seizure after chronic subdural hematoma evacuation: associated factors and effect on clinical outcome

**DOI:** 10.3389/fneur.2023.1190878

**Published:** 2023-05-09

**Authors:** Liang Wu, Xufei Guo, Yunwei Ou, Xiaofan Yu, Bingcheng Zhu, Yunfei Li, Weiming Liu

**Affiliations:** ^1^Department of Neurosurgery, Beijing Tiantan Hospital, Capital Medical University, Beijing, China; ^2^Beijing Neurosurgical Institute, Capital Medical University, Beijing, China; ^3^China National Clinical Research Center for Neurological Diseases, Beijing, China; ^4^Beijing Advanced Innovation Center for Big Data-Based Precision Medicine, Beihang University, Beijing, China; ^5^Neurological Center, People’s Hospital of Ningxia Hui Autonomous Region (The Third Clinical Medical College, Ningxia Medical University), Yinchuan, China

**Keywords:** chronic subdural hematoma, postoperative seizure, burr-hole craniotomy, recurrence, clinical outcome

## Abstract

**Objective:**

Chronic subdural hematoma (CSDH) is a common disease in neurosurgery, which usually occurs in the elderly. Seizure is one of the postoperative complications in CSDH patients and can affect patient outcomes. There is currently no consensus on whether antiepileptic drugs should be prescribed prophylactically. The aim of this study was to evaluate independent risk factors for postoperative seizures and unfavorable outcomes in CSDH patients.

**Methods:**

We reviewed 1,244 CSDH patients who had undergone burr-hole craniotomy in this study. Patient clinical data, CT scan results, recurrence and outcome data were collected. We divided the patients into two groups based on whether they had a postoperative seizure. Percentages and *χ*^2^ tests were applied for categorical variables. Standard deviations and two-sided unpaired *t*-tests were applied for continuous variables. Stepwise logistic regression analyses were performed to identify the independent factors of postoperative seizures and unfavorable outcomes.

**Results:**

The incidence of seizures after CSDH surgery was 4.2% in this study. There was no significant difference in recurrence rate between seizure and non-seizure patients (*p* = 0.948), and the outcome of seizure patients was significantly poor (*p* < 0.001). There are more postoperative complications in seizure patients (*p* < 0.001). Logistic regression analysis showed that the independent risk factors for postoperative seizures included drinking history (*p* = 0.031), cardiac disease (*p* = 0.037), brain infarction (*p* = 0.001) and trabecular hematoma (*p* < 0.001). The use of urokinase is a protective factor for postoperative seizures (*p* = 0.028). Hypertension is an independent risk factor for unfavorable outcome in seizure patients (*p* = 0.038).

**Conclusion:**

Seizures after CSDH surgery were associated with postoperative complications, higher mortality and poorer clinical outcomes at follow-up. We believe that alcohol consumption, cardiac disease, brain infarction and trabecular hematoma are independent risk factors for seizures. The use of urokinase is a protective factor against seizures. Patients with postoperative seizures need more stringent management of their blood pressure. A prospective randomized study is necessary to determine which subgroups of CSDH patients would benefit from antiepileptic drugs prophylaxis.

## 1. Introduction

Chronic subdural hematoma (CSDH) is one of the most common diseases in neurosurgery, especially in the elderly, which is characterized by the blood collection wrapped by the inner and outer membranes (1). Currently, the incidence of CSDH is about 7.2–10.3 per 100,000 people and is expected to increase to 17.1 per 100,000 by 2030 (2). In China, as the population ages, the incidence of CSDH is also gradually increasing (3, 4). At present, the pathological mechanism of CSDH is not clear. As a classical hypothesis, it has been widely cited that head injury leads to tearing and bleeding of the bridging vein between the brain and the dura mater. Alternatively, it has been hypothesized that the separation of dural boundary cells and the formation of subdural effusion are the initial factors leading to CSDH (1, 5).

As the disease progresses, increased CSDH can lead to compression of the cerebral parenchyma, and can cause headaches, nausea, limb movement disorders, unconsciousness, and focal neurological deficits (6). Treatment for CSDH consists mainly of surgical treatment and non-surgical treatment (7). At present, burr-hole craniotomy is the standard treatment for most patients with CSDH. It is simple, safe, and less invasive, even for patients over the age of 90 (8, 9). As one of the post-surgery complications of CSDH, seizures are not common in the clinic, as the patient’s cerebral parenchyma is normal in most cases (1). However, postoperative seizures can adversely affect the rehabilitation of patients, and even lead to increased mortality (10–14). Seizures may occur in association with occult cortical injury, decreased cerebral blood flow, and hemoglobin degradation products (5, 10, 15). There is also controversy about the need for prophylactic antiepileptic drugs (AEDs) in CSDH patients (1, 2, 16, 17).

The purpose of this study was to retrospectively analyze the incidence of postoperative seizures and functional outcomes in CSDH patients receiving standardized surgical treatment in a single institution. In addition, we used stepwise logistic regression to identify independent risk factors for postoperative seizures and adverse outcomes in seizure patients.

## 2. Materials and methods

### 2.1. Study population

We reviewed the continuous treatment of CSDH patients at Beijing Tiantan Hospital, Capital Medical University from November 2011 to July 2019. At our medical center, surgical intervention is commonly performed for symptomatic subdural hematomas, which are commonly characterized by confirmed CT findings and patients with obvious neurological symptoms such as headache, dizziness, limb weakness, dysphasia, and seizures. A total of 1,360 patients were recruited, of whom 1,244 were treated surgically and the remaining 116 treated conservatively were excluded from the study. All enrolled patients underwent burr-hole craniotomy and postoperative exhaustive drainage, as detailed in our previous report (18). By reviewing previous clinical records, we identified 52 patients with postoperative seizures, with an incidence of 4.2%. Due to limitations in medical records, we only recorded seizure events without distinguishing in detail the type and timing of the seizures. Ultimately, all patients who underwent surgery and enrolled in the study were divided into two groups (with seizures: without seizures = 52: 1192; Figure 1).

**Figure 1:**
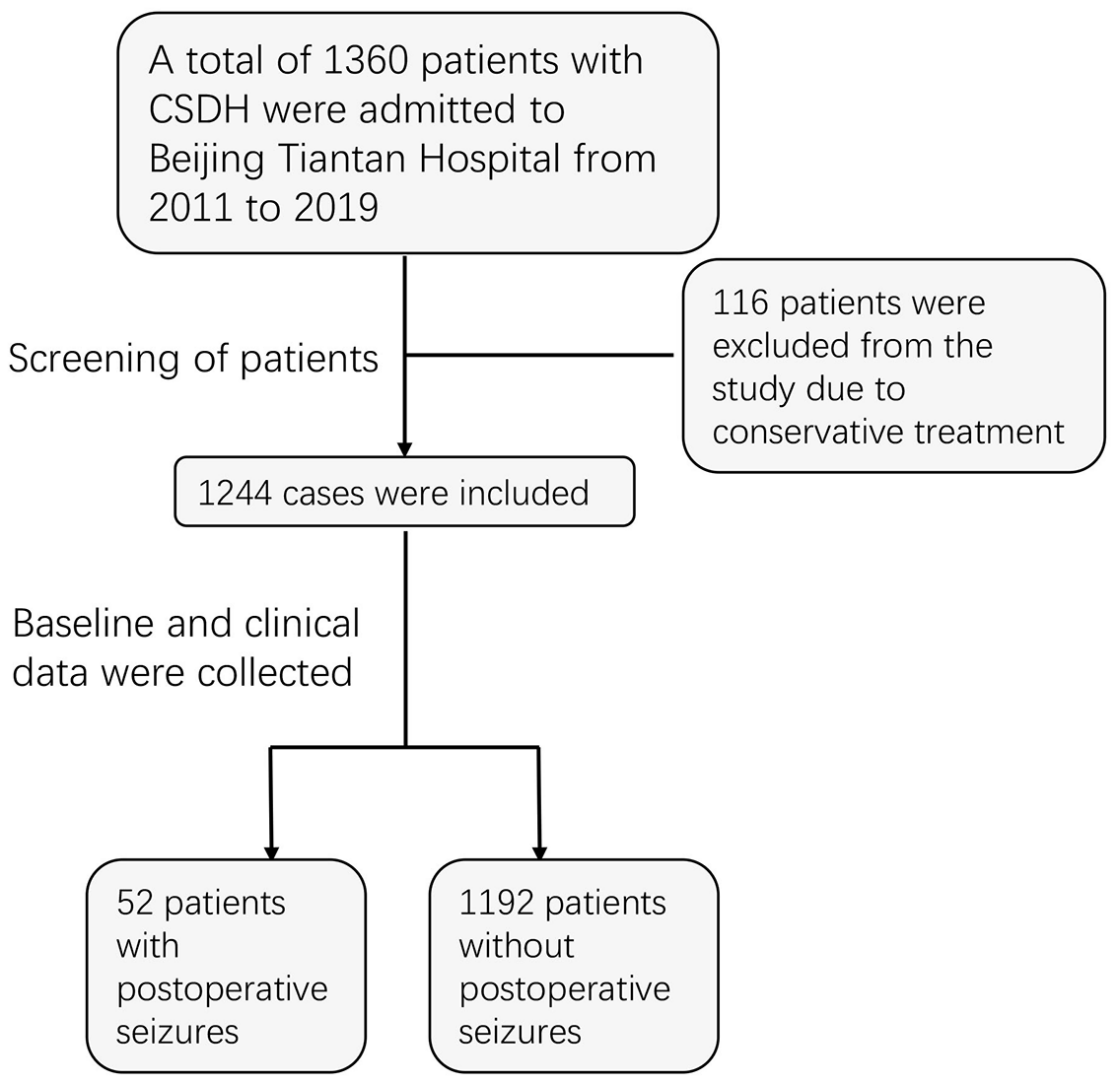
Flow chart of patient screening and inclusion.

### 2.2. Date collection

The following characteristics of all enrolled patients were collected, including gender, age, history of trauma, preoperative symptoms, personal history, and history of anticoagulant drug use. All patients should stop taking anticoagulants for at least 7 days before surgery. Preoperative coagulation tests included international standardized ratio (INR), activated partial thromboplastin time (APTT), prothrombin time (PT), thrombin time (TT) and fibrinogen (Fg). An abnormality in any of the indices is considered as a dysfunction of the coagulation. In addition, the laterality of the hematoma and the pre/postoperative maximum transverse diameter of the hematoma were determined from CT scans. The reduction of the hematoma is calculated from the reduction of the transverse diameter after the operation. According to the suggestion by Milo et al. (19), hematoma is divided into 7 types (type A: hypodense; type B: isodense; type C: hyperdense; type D: laminar; type E: separated; type F: gradation; type G: trabecular). We documented in detail the postoperative complications of the patients, including pneumonia, rebleeding, thrombotic events, coma, wound infection, and death. Two neurosurgeons independently followed up patients who had been discharged from the hospital for more than 6 months by telephone to track their outcomes. We used the modified Rankin score to evaluate the neurological function outcomes and quality of life of the patients. We define a score of 0–3 as favorable and a score of 4–6 as unfavorable. CSDH recurrence is defined as the hematoma reaccumulation in the hematoma cavity and the preoperative symptoms reoccurrence within 6 months. This study was approved by the Institutional Review Board of Beijing Tiantan Hospital, Capital Medical University (Ethical inspection No. KY2020-094-02). Written informed consent was obtained from all enrolled patients.

### 2.3. Statistical analyses

We used IBM SPSS Statistics (version 26.0; IBM Corp.) to analyze the data. The data for the categorical variables were presented as numbers and percentages, and the differences between groups were compared by chi-squared tests. Continuous variables were expressed as mean and standard deviation, and the differences between groups were compared by independent t-tests. Forward stepwise logistic regression analysis was used to identify the independent risk factors for postoperative seizures and unfavorable outcomes. All tests were double-tailed, and *p* < 0.05 was considered to be significant.

## 3. Results

### 3.1. Preoperative clinical features among CSDH patients

Our study included a total of 1,244 patients, 52 in the seizure group and 1,192 in the non-seizure group. As shown in Table 1, there was no significant difference in the proportion of males between the two groups, which were 84.6 and 82.5%, respectively. With regard to age, the average age of patients with postoperative seizures was 65.08 ± 14.64 years old, while that of patients without seizures was 63.03 ± 16.98 years old, and the difference did not reach statistical significance. Among the symptoms on admission, the proportion of preoperative dysphasia in patients with postoperative seizures was higher than that in patients without seizures (*p* = 0.043). As for the comorbidities of patients, the proportions of previous drinking (*p* = 0.004), cardiac disease (*p* = 0.011) and brain infarction (*p* < 0.001) in the postoperative seizures group were higher than that in the non-seizures group. There were no significant differences between the two groups in smoking history, hypertension and diabetes. In addition to the above, there were no significant differences between the two groups in terms of trauma history, anticoagulant use, coagulation function on admission, and the laterality as well as maximum transverse diameter of the hematoma. It is worth noting that there is a great difference in the type of hematoma between the two groups, and the probability of trabecular hematoma in the postoperative seizures group is considerably higher than that in the non-seizures group (36.5 vs. 15.7%, *p* < 0.001).

**Table 1:** Preoperative clinical features among chronic subdural hematoma (CSDH) patients with or without seizures after surgery.

Characteristics analyzed	Postoperative seizures (*n*)		*p*
	Yes (52)	No (1,192)	
Gender (Male:Female)	44:8	984:208	0.700
Age	65.08 ± 14.64	63.03 ± 16.98	0.393
Symptoms			
Headache *n* (%)	28 (53.8)	701 (58.8)	0.477
Dizziness *n* (%)	10 (19.2)	323 (27.1)	0.210
Limb weakness *n* (%)	31 (59.6)	659 (55.3)	0.539
Dysphasia *n* (%)	9 (17.3)	107 (9.0)	0.043
Disturbance of consciousness *n* (%)	3 (5.8)	57 (4.8)	0.745
Personal/past history			
Smoking	18 (34.6)	282 (23.7)	0.071
Drinking	17 (32.7)	205 (17.2)	0.004
Hypertension	20 (38.5)	423 (35.5)	0.661
Diabetes	13 (25.0)	241 (20.2)	0.402
Cardiac disease	6 (11.5)	49 (4.1)	0.011
Brain infarction	17 (32.7)	132 (11.1)	<0.001
Head trauma event *n* (%)	36 (69.2)	747 (62.7)	0.337
History of anticoagulant drug	7 (13.5)	132 (11.1)	0.593
Preoperative dysfunction of coagulation	15 (28.8)	365 (30.6)	0.786
Unilateral/bilateral hematoma			0.860
Left	22(42.3)	490(41.1)	
Right	18(34.6)	387(32.5)	
Bilateral	12(23.1)	315(26.4)	
Types of hematoma			0.001
A	13 (25.0)	198 (16.6)	
B	7 (13.5)	352 (29.5)	
C	6 (11.5)	262 (22.0)	
D	3 (5.8)	89 (7.5)	
E	1 (1.9)	59 (4.9)	
F	3 (5.8)	45 (3.8)	
G	19 (36.5)	187 (15.7)	
Trabecular hematoma	19 (36.5)	187 (15.7)	< 0.001
Maximum transverse diameter of hematoma in cm	2.11 ± 0.82	2.20 ± 0.65	0.371

### 3.2. Postoperative clinical features among CSDH patients

As shown in Table 2, the reduction of hematoma volume after operation in the seizures group was lower than that in the non-seizures group (*p* = 0.017). However, there was no significant difference between the two groups in the use and frequency of postoperative urokinase. The duration of drainage catheter in the seizures group was slightly longer than that in the non-seizures group (*p* = 0.037). In addition, the incidence of postoperative complications in the seizures group was significantly higher than that in the non-seizures group (*p* < 0.001). As shown in Table 3, postoperative coma (*p* < 0.001), pneumonia (*p* = 0.012), and mortality (*p* < 0.001) in the seizures group were higher than those in the non-seizures group.

**Table 2:** Postoperative clinical features among CSDH patients with or without seizures after surgery.

Characteristics Analyzed	Postoperative seizures (*n*)		*p*
	Yes (52)	No (1192)	
Reduction of hematoma cavity (%)	53.5 ± 18.4	60.9 ± 21.0	0.017
Duration of drainage catheter (day)	3.9 ± 1.6	3.3 ± 1.9	0.037
Use of urokinase	21 (40.3)	577 (48.4)	0.257
Frequency of urokinase used	0.83 ± 1.06	0.91 ± 1.06	0.579
Complications	13 (25.0)	59 (4.9)	<0.001
Recurrence requiring reoperation	2 (3.8)	48 (4.0)	0.948
Outcomes (mRS)			<0.001
0–3	46 (88.4)	1,169 (98.0)	
4–6	6 (11.5)	23 (1.9)	

mRS, modified Rankin Scale.

**Table 3:** Complications among CSDH patients with or without seizures after surgery.

Complications	Seizures (52)	Non-seizures (1192)	*p*
Thrombotic events	1 (1.9)	4 (0.3)	0.076
Fever	1 (1.9)	32 (2.7)	0.740
Wound infection	0	3 (0.2)	0.717
Coma	4 (7.6)	3 (0.2)	<0.001
Pneumonia	2 (3.8)	8 (0.6)	0.012
Rebleeding	1 (1.9)	5 (0.4)	0.131
Death	4 (7.6)	4 (0.3)	<0.001

From our results, postoperative seizures seemed to have no effect on the recurrence of hematoma (*p* = 0.948), and the recurrence rates in the two groups were 3.8 and 4.0%, respectively. However, we found that the outcomes of patients in the seizures group were worse than that in the non-seizures group, and the incidence of unfavorable outcomes in the seizures group was higher (*p* < 0.001).

### 3.3. Independent risk factors for postoperative seizures

We screened out the independent risk factors for postoperative seizures in CSDH patients through a stepwise logistic regression analysis. As presented in Table 4, after including the relevant variables of all patients before and after operation, we found that drinking history (*p* = 0.031, *B* = 0.735, Exp (B) = 2.085, 95% CI 1.072–4.057), cardiac disease (*p* = 0.037, *B* = 1.048, Exp (B) = 2.852, 95% CI 1.066–7.634), brain infarction (*p* = 0.001, *B* = 1.134, Exp (B) = 3.108, 95% CI 1.588–6.080) and trabecular hematoma (*p* < 0.001, *B* = 1.344, Exp (B) = 3.834, 95% CI 2.008–7.321) were independent risk factors for postoperative seizures. In addition, we also found that the use of urokinase is a protective factor against postoperative seizures (*p* = 0.028, *B* = −0.707, Exp (B) = 0.493, 95% CI 2.262–0.927).

**Table 4:** Forward stepwise logistic analyses of factors related to postoperative seizures in CSDH patients.

Variable	*B*	SE	Wald test	*p*	Exp (B)	OR (95%CI)
Step 1 Trabecular hematoma	1.194	0.308	14.996	<0.001	3.301	1.804–6.042
Constant	−3.432	0.192	319.413	<0.001	0.032	
Step 2 Brain infarction	1.163	0.331	12.308	<0.001	3.199	1.671–6.127
Trabecular hematoma	1.139	0.312	13.349	<0.001	3.125	1.696–5.758
Constant	−3.658	0.215	290.742	<0.001	0.026	
Step 3 Cardiac disease	1.150	0.494	5.412	0.020	3.157	1.199–8.318
Brain infarction	1.063	0.338	9.873	0.002	2.896	1.492–5.621
Trabecular hematoma	1.122	0.314	12.790	<0.001	3.073	1.661–5.684
Constant	−3.711	0.218	289.568	<0.001	0.024	
Step 4 Cardiac disease	1.011	0.502	4.053	0.044	2.749	1.027–7.358
Brain infarction	1.083	0.342	10.040	0.002	2.953	1.511–5.769
Trabecular hematoma	1.306	0.329	15.766	<0.001	3.690	1.937–7.030
Use of urokinase	−0.687	0.322	4.554	0.033	0.503	0.268–0.946
Constant	−3.413	0.246	192.617	<0.001	0.033	
Step 5 Drinking	0.735	0.340	4.680	0.031	2.085	1.072–4.057
Cardiac disease	1.048	0.502	4.355	0.037	2.852	1.066–7.634
Brain infarction	1.134	0.342	10.964	0.001	3.108	1.588–6.080
Trabecular hematoma	1.344	0.330	16.583	<0.001	3.834	2.008–7.321
Use of urokinase	−0.707	0.322	4.821	0.028	0.493	0.262–0.927
Constant	−3.611	0.272	175.630	<0.001	0.027	

CI, confidence interval.

### 3.4. Independent risk factors for unfavorable outcomes

As shown in Table 5, we explored independent risk factors for unfavorable outcomes after including all CSDH patients in the stepwise logistic regression analysis. In the end, a total of four variables had a significant impact on the adverse outcomes of patients: age (*p* = 0.004, *B* = 0.063, Exp (B) = 1.065, 95% CI 1.020–1.112), hypertension (*p* = 0.005, *B* = 1.305, Exp (B) = 3.687, 95% CI 1.491–9.117), brain infarction (*p* = 0.001, *B* = 1.435, Exp (B) = 4.199, 95% CI 1.838–9.591), and postoperative complications (*p* < 0.001, *B* = 2.071, Exp (B) = 7.932, 95% CI 3.258–19.313). For patients in the postoperative seizures group, the results showed that only hypertension (*p* = 0.038, *B* = 2.377, Exp (B) = 10.769, 95% CI 1.140–101.721) was an independent risk factor for adverse outcomes. The results are shown in Table 6.

**Table 5:** Forward stepwise logistic analyses of factors related to outcomes in CSDH patients.

Variable	*B*	SE	Wald test	*p*	Exp (B)	OR (95%CI)
Step 1 Complications	2.252	0.419	28.823	<0.001	9.507	4.178–21.631
Constant	−4.077	0.245	277.796	<0.001	0.017	
Step 2 Brain infarction	1.817	0.410	19.652	<0.001	6.151	2.755–13.733
Complications	2.137	0.436	24.006	<0.001	8.475	3.605–19.926
Constant	−4.552	0.305	222.378	<0.001	0.011	
Step 3 Hypertension	1.451	0.459	9.969	0.002	4.265	1.733–10.495
Brain infarction	1.632	0.417	15.112	<0.001	5.066	2.236–11.481
Complications	2.130	0.445	22.904	<0.001	8.412	3.517–20.122
Constant	−5.301	0.445	142.214	<0.001	0.005	
Step 4 Age	0.063	0.022	8.177	0.004	1.065	1.020–1.112
Hypertension	1.305	0.462	7.983	0.005	3.687	1.491–9.117
Brain infarction	1.435	0.421	11.591	0.001	4.199	1.838–9.591
Complications	2.071	0.454	20.803	<0.001	7.932	3.258–19.313
Constant	−9.627	1.694	32.293	<0.001	<0.001	

CI, confidence interval.

**Table 6:** Forward Stepwise Logistic analyses of factors related to outcomes in CSDH patients with postoperative seizures.

Variable	*B*	SE	Wald test	*p*	Exp (B)	OR (95%CI)
Step 1 Hypertension	2.377	1.146	4.303	0.038	10.769	1.140–101.721
Constant	−3.332	1.018	10.721	0.001	0.036	

CI, confidence interval.

## 4. Discussion

Chronic subdural hematoma is one of the most common conditions faced by neurosurgeons worldwide. As a disease that primarily occurs in elderly patients, with the aging of the population, CSDH is becoming more and more common in clinical practice (20). Postoperative seizure is a potential complication after surgical removal of CSDH. The incidence of postoperative seizures reported in the literature varies widely, ranging from 0.67 to 32.0% (13, 21). Our study reported 4.2% of the incidence (52/1244), which is within the scope of the existing literature. The reasons for such a large difference are various. There are several reasons for such a wide variation in seizures rates among the studies. The definition of seizure, the population included, the choice of surgical protocol, and the use of prophylactic AEDs may all affect incidence. The reported incidence in studies without routine use of EEG monitoring tends to be low, between 0.7 and 5.4% (1). Nayil et al. (22) included 1,181 patients, of whom only 8 (0.7%) had seizures.

Our results suggest that a history of alcohol consumption, cardiac disease, brain infarction, and the trabecular hematoma present on preoperative CT are independent risk factors for postoperative seizures. However, in our study, we did not find that gender, age, trauma history, anticoagulant use, unilateral or bilateral hematoma, and maximum transverse diameter of hematoma had any effect on postoperative seizures.

Risk factors for seizures after CSDH surgery have been reported in the literature. Hussam et al. (5) did a literature overview in their study summarizing findings from previous articles, including mixed density hematoma, right-side hematoma, female sex, midline shift, membranectomy, and depressed brain volume. Alcoholism and remote stroke have also been reported as risk factors for postoperative seizures. In our study, the proportion of postoperative seizure patients with a history of alcohol abuse reached 32.7%. The incidence of postoperative seizures in the 131 patients included by Kotwica et al. (23) was 7%, among whom 45 (34%) had a history of alcoholism. Rubin et al. (24) believe that alcoholics often have repeated head injuries that lead to brain damage. In addition, there are various mechanisms that reduce the threshold for seizures in alcohol abuse patients. These increased risks include brain atrophy, changes in certain neurotransmitter pathway systems, and ionic or metabolic imbalances (25). Similar to the results of Won et al. (15), we also found that patients with a history of stroke had a higher incidence of seizures. In our study, 17 (32.7%) patients with postoperative seizures had a previous history of cerebral infarction. In patients with previous brain infarction, there are some permanent changes in the central nervous system that lead to the emergence of potential epileptogenic foci. These changes include gliosis within the cortex and loss of brain tissue, resulting in the destruction of neural networks and the increase of neuronal excitability (26). In our study, a higher proportion of postoperative seizure patients had complicated cardiac disease than those without seizures. Patients with a previous history of coronary heart disease may experience arrhythmias and insufficient cardiac output under the shock of surgery, which can affect blood supply to the brain and induce seizures. Mixed density hematoma as a risk factor for seizures in CSDH has been confirmed by many studies (10, 11, 27), which has also been verified in our data. In our study, trabecular hematoma accounted for a large proportion of postoperative seizures patients, twice as numerous as non-seizures patients (36.5 vs. 15.7%). This may be due to the stimulating effect of hemoglobin and fibrin and their breakdown products on the cerebral cortex.

Unlike previous studies, we were surprised to find a protective effect of urokinase against postoperative seizures. Although multivariate analysis showed no difference, in univariate analysis, we found that the reduction of hematoma cavity in patients with postoperative seizures was lower than in patients without seizures (53.5 vs. 60.9%). At our medical center, we routinely use urokinase to promote the drainage of hematoma fluid in postoperative CSDH patients. Therefore, the possible reason for this difference is that non-seizure patients receive more urokinase and therefore have fewer residual hematomas than those who have seizures, and the more residual hematomas, the higher the risk of seizures, since more seizure-inducing substances remain.

Currently, it is commonly believed that postoperative seizure is an independent predictor of adverse outcomes (15, 20). Postoperative seizures in CSDH patients may be associated with significant complications, prolonged hospital stays, and poor functional outcomes (27). Our study did not show a relationship between postoperative seizures and CSDH recurrence, but patients with seizures had more postoperative complications and higher mortality during hospitalization. At the same time, the proportion of seizure patients with unfavorable outcomes was also higher than non-seizure patients (11.5 vs. 1.9%).

There is still controversy about the prophylactic use of AEDs in CSDH patients, and the 2013 Cochrane review failed to provide meaningful conclusions (16). A meta-analysis done by Deivanai et al. (1) did not show any significant reduction in seizure risk in CSDH patients after taking AEDs. At our center, AEDs are not a routine option after surgery due to the low incidence of postoperative seizures, but are commonly used in patients who do have symptomatic seizures. The selection of AEDs is often based on the practice of the physician in charge. Phenobarbital, levetiracetam, and valproate are routine options. At the same time, since we only studied postoperative seizures during hospitalization and did not provide long-term follow-up data, we cannot determine whether prophylactic use of AEDs is beneficial. Given the poor prognosis of seizure patients, prophylactic use of AEDs may be necessary, but further prospective studies are needed to validate this. Studies using subgroup analysis and risk scores are necessary to determine which specific populations can benefit (27). In addition, our study analyzed risk factors for adverse outcomes in CSDH patients. In contrast to all CSDH patients, whose risk factors for adverse outcomes included age, hypertension, brain infarction, and postoperative complications, hypertension was the only risk factor for seizure patients. Therefore, blood pressure management is also extremely critical in postoperative patients with seizures, and proper blood pressure control may be able to improve the patient outcomes.

## 5. Limitation

Because this study is a retrospective, single-center study, the determination of postoperative seizures is based on previous medical records, and not all patients with CSDH have received intensive care after surgery, so some seizure patients may not be detected and recorded, which affects the accuracy of reported incidence of postoperative seizures. We also did not record the patient’s past history of epilepsy, and these patients may have a higher risk of postoperative seizures. In addition, we assessed the risk of seizures only during hospitalization, and we did not provide long-term follow-up data for patients with seizures. Finally, it is unclear whether AEDs prophylaxis will translate into clinical benefits for CSDH patients. More prospective observational or interventional studies are needed to help clinicians make decisions in the future.

## 6. Conclusion

Seizures after CSDH surgery were associated with postoperative complications, higher mortality and poorer clinical outcomes at follow-up. We believe that alcohol consumption, cardiac disease, brain infarction and trabecular hematoma are independent risk factors for seizures. The use of urokinase is a protective factor against seizures. Patients with postoperative seizures need more stringent management of their blood pressure. A prospective randomized study is necessary to determine which subgroups of CSDH patients would benefit from AEDs prophylaxis.

## Data availability statement

The raw data supporting the conclusions of this article will be made available by the authors, without undue reservation.

## Ethics statement

The studies involving human participants were reviewed and approved by Institutional Review Board of Beijing Tiantan Hospital, Capital Medical University (Ethical inspection No. KY2020-094-02). Written informed consent to participate in this study was provided by the participants’ legal guardian/next of kin.

## Author contributions

WL conceived and designed the study. LW and XG contributed to data collection and analysis, and wrote the original draft of the manuscript. YO and YL provided the technical support. BZ and XY revised and finalized the manuscript. All authors contributed to the article and approved the submitted version.

## Funding

This work was supported by the Capital’s Funds for Health Improvement and Research (2020-2-2045).

## Conflict of interest

The authors declare that the research was conducted in the absence of any commercial or financial relationships that could be construed as a potential conflict of interest.

## Publisher’s note

All claims expressed in this article are solely those of the authors and do not necessarily represent those of their affiliated organizations, or those of the publisher, the editors and the reviewers. Any product that may be evaluated in this article, or claim that may be made by its manufacturer, is not guaranteed or endorsed by the publisher.
